# Prediction Model for Detecting Active Pulmonary Tuberculosis in Patients With Head and Neck Cancer Prior to Concurrent Chemoradiotherapy

**DOI:** 10.14740/jocmr6634

**Published:** 2026-09-26

**Authors:** Chawalit Lakdee, Jayanton Patumanond, Peeraphong Thiarawat, Thanin Lokeskrawee, Suppachai Lawanaskol, Pornpit Treebupachatsakul, Wanwisa Bumrungpagdee, Suwapim Chanlaor

**Affiliations:** a Department of Radiology, Buddhachinaraj Phitsanulok Hospital, Phitsanulok, Thailand; b Clinical Epidemiology and Clinical Statistics Unit, Faculty of Medicine, Naresuan University, Phitsanulok, Thailand; c Department of Surgery, Faculty of Medicine, Naresuan University, Phitsanulok, Thailand; d Department of Emergency Medicine, Lampang Hospital, Lampang, Thailand; e Chaiprakarn Hospital, Chiang Mai, Thailand; f Department of Medicine, Buddhachinaraj Phitsanulok Hospital, Phitsanulok, Thailand

**Keywords:** Logistic models, Body mass index, Hemoglobin, Serum albumin, Diagnosis

## Abstract

**Background:**

Patients with cancer, particularly those with head and neck cancer (HNC), have a higher risk of developing active pulmonary tuberculosis (PTB) than the general population. Radiotherapy or concurrent chemoradiotherapy (CCRT) is a primary treatment modality for HNC. Therefore, the detection of active PTB before treatment is important. However, routine testing in all patients is not feasible. We aimed to develop a diagnostic prediction model for active PTB using routinely available pretreatment clinical and laboratory parameters in patients with HNC prior to radiotherapy.

**Methods:**

We performed a diagnostic prediction study with a retrospective, cross-sectional design using medical record reviews of all patients diagnosed with HNC who underwent radiotherapy at the Radiation Therapy Unit, Buddhachinaraj Phitsanulok Hospital, Phitsanulok, Thailand, from October 2014 to October 2025. Active PTB was defined as the initiation of anti-tuberculosis treatment with microbiological confirmation (acid-fast bacilli smear microscopy, culture, or nucleic acid amplification testing/GeneXpert) and/or chest imaging compatible with active PTB. Multivariable logistic regression was performed to derive the models. Discrimination was measured using the area under the receiver operating characteristic curve (AuROC); calibration was assessed using calibration plots, and internal validation was performed using bootstrap resampling. Decision curve analysis was used to determine clinical utility.

**Results:**

Of the 505 patients with HNC, 30 (5.94%) had active PTB. Four predictors independently associated with active PTB were body mass index (BMI) (odds ratio (OR) 0.86; 95% confidence interval (CI) 0.75–0.99), hemoglobin (OR 0.75; 95% CI 0.58–0.95), albumin (OR 0.25; 95% CI 0.12–0.53), and cancer symptom duration (months) (OR 1.49; 95% CI 1.25–1.76). The model showed excellent discrimination (AuROC 0.882; 95% CI 0.814–0.950) and good calibration (slope 1.000). At a predicted probability cutoff (≥ 5.94%), sensitivity was 90.0% (95% CI 73.5–97.9) and specificity was 82.3% (95% CI 78.6–85.6). The model may reduce the number of patients requiring confirmatory PTB testing, missing only three of 505 patients (0.6%) overall.

**Conclusions:**

In patients with HNC undergoing pretreatment evaluation, a simple four-variable model (BMI, hemoglobin level, albumin concentration, and cancer symptom duration) can estimate the probability of active PTB.

## Introduction

In 2024, an estimated 10.7 million people fell ill with tuberculosis (TB) worldwide, of which pulmonary TB (PTB) accounted for approximately 84% of newly diagnosed and relapsed cases [1]. Thailand is an endemic country for TB, and the World Health Organization (WHO) estimates the incidence of TB to be 157 per 100,000 population [2]. According to a systematic review and meta-analysis, the incidence of TB in patients with cancer is higher than that in the general population [3].

In a population-based retrospective cohort study, patients with head and neck cancer (HNC) had a 2.86-fold higher incidence of PTB than those without cancer [4]. Patients with HNC are at an increased risk of active PTB, a susceptibility that may be explained by impaired host defense and common respiratory risk factors [4]. In a retrospective series of patients with squamous cell carcinoma (SCC) HNC, clinically relevant findings (active PTB, sequelae, or pneumonia) were observed in 6.6% of cases [5]. The risk of TB is higher at the time of cancer diagnosis (the year before and after diagnosis), highest in the first 6 months, and persists beyond 24 months compared to that in the general population [6, 7]. Cancer-associated immunosuppression may increase the risk of PTB. Additional risk factors include surgical procedures, such as splenectomy and appendectomy, and comorbidities that are known to depress immune function [8, 9]. Undernutrition is a major risk factor for deterioration from *Mycobacterium tuberculosis* infection to active TB disease and is highly prevalent at the time of diagnosis, with a prevalence rate of 40.7% in one multicenter hospital [10]. Advanced age, male sex, and specific cancer sites are independent risk factors for TB among cancer patients in TB-endemic settings [11]. Previous studies have predominantly focused on developing PTB prediction models for the general population and hospital inpatients, utilizing clinical symptoms and radiographic findings [12–16]. Since HNC management requires intensive multidisciplinary team (MDT) care [17], timely diagnosis is critical not only for patient outcomes but also for infection control. Healthcare workers are already at an increased risk of active TB compared with the general population [18].

However, no studies have focused on developing simple clinical prediction models to aid in the diagnosis of active PTB specifically in patients with HNC. The objective of this study was to develop a diagnostic prediction model for active PTB in patients with HNC undergoing pretreatment evaluation, using clinical and laboratory parameters that are routinely available at the time of pretreatment workup and require no additional investigations.

## Materials and Methods

### Study design

We conducted a diagnostic prediction study with a retrospective observational cross-sectional design by reviewing medical records of adults (aged 18 years or older) with stage I to IV (M0) head and neck SCC involving the glottis, oropharynx, oral cavity, hypopharynx, or nasopharynx. Eligible patients underwent pretreatment workup at the Radiation Therapy Unit, Buddhachinaraj Phitsanulok Hospital, Thailand, and had an indication for postoperative radiotherapy or definitive concurrent chemoradiotherapy (CCRT) between October 2014 and October 2025. Tumors were staged according to the American Joint Committee on Cancer (AJCC) staging system. Patients were excluded if they had prevalent active TB and had started anti-TB treatment before the date of cancer diagnosis, or if they withdrew before treatment initiation.

### Participant and data collection

#### Participants

Pretreatment clinical data were obtained from medical records of patients with histopathologically confirmed HNC whose first visit to the Radiation Therapy Unit occurred during the pretreatment phase. Patients diagnosed with TB before cancer staging was completed were excluded. Disease assessment was conducted using computed tomography (CT), magnetic resonance imaging (MRI), or bone scintigraphy and was defined as complete staging. The variables collected were cancer symptom duration (months), demographics (sex, age, and body mass index (BMI, kg/m^2^)), comorbidities (diabetes mellitus, hypertension, dyslipidemia), lifestyle factors (smoking, alcohol consumption), tumor characteristics (primary site, histologic grade, tumor staging T1 to T4, nodal staging N0 to N3, maximum tumor diameter, maximum lymph node diameter), laboratory parameters (hemoglobin, albumin, neutrophil-to-lymphocyte ratio), and human papillomavirus (HPV) status. We referred patients for further investigation and microbiological confirmation when CT staging suggested active PTB.

Comorbidities were defined pharmacologically: diabetes mellitus, hypertension, and dyslipidemia were each recorded as present if the patient was receiving pharmacotherapy for that condition at the time of the pretreatment assessment, and as absent otherwise. Lifestyle factors were recorded as binary indicators of ever-exposure as documented in the medical record: smoking history (current or former smoker versus never) and alcohol consumption (current or former drinker versus never). Quantitative measures of alcohol intake or smoking exposure, such as volume, frequency, duration, or pack-years, were not available from the medical records.

#### Endpoints

Active PTB was defined as initiation of anti-TB treatment together with at least one supporting finding: microbiological confirmation (acid-fast bacilli (AFB) smear microscopy, mycobacterial culture, or nucleic acid amplification testing (NAAT/GeneXpert)), chest imaging compatible with active PTB, or histopathological evidence when available [15, 19].

#### Specific time points for prediction

Pretreatment clinical data collected at the first visit to the Radiation Therapy Unit before completion of staging were used to predict active PTB. Patients with findings suggestive of active PTB during staging were referred for further investigation and microbiological confirmation of the diagnosis.

#### Study size estimation

The sample size was determined using Stata (version 17.0), with assumptions based on a pilot study with four candidate predictors, an expected C-statistic of 0.80, and an estimated active PTB prevalence of 0.059. This calculation revealed the least required sample size of 473 participants, which equated to approximately 28 active PTB events. The estimated prevalence was obtained from a review of medical records at the Radiation Therapy Unit of Buddhachinaraj Phitsanulok Hospital.

### Statistical analysis

Statistical analyses were performed using Stata version 17.0. Categorical variables are presented as frequencies and percentages and were compared using Fisher’s exact test. Continuous variables are presented as mean ± standard deviation (SD) for approximately normally distributed data and compared using the Student’s *t*-test; non-normally distributed variables are presented as median (interquartile range (IQR)) and compared using the Wilcoxon rank-sum test. Missing data were handled using multiple imputations. Sensitivity analyses comparing multiple imputations with complete-case analysis showed no meaningful differences.

### Model derivation

This study was designed as a diagnostic prediction study rather than an etiologic study. Accordingly, no single variable was designated as the primary exposure of interest, and variables were not selected on the basis of a causal confounding framework.

Four pretreatment predictors were pre-specified on clinical grounds before model fitting, rather than by data-driven or automated selection: BMI, hemoglobin concentration, serum albumin concentration, and cancer symptom duration. These variables were chosen because together they characterize the nutritional and inflammatory status through which host susceptibility to progression from latent infection to active PTB is thought to operate, and because all four are routinely recorded during pretreatment workup and were therefore available at the intended time of prediction. All four variables were entered simultaneously into a multivariable logistic regression model and were retained in the final model.

The multivariable odds ratio (mOR) for each predictor therefore represents its conditional association with active PTB given the other three predictors in the model. Because the objective was prediction rather than causal inference, the regression coefficients were interpreted primarily as predictive weights contributing to the estimated probability of active PTB rather than as causal effect estimates. Multicollinearity among the predictors was assessed before model fitting, with no evidence of collinearity. The remaining pretreatment variables described above were examined descriptively and in univariable analyses but were not entered into the multivariable model, in keeping with the pre-specified approach and with the number of predictor parameters supported by the sample size estimation described above.

Discrimination was evaluated using the area under the receiver operating characteristic curve (AuROC) and the 95% confidence interval (CI). A calibration plot, calibration slope, expected-to-observed (E:O) ratio, and calibration-in-the-large (CITL) were used to evaluate the calibration. Internal validation was performed using bootstrap resampling (200 repetitions). The clinical utility was determined using decision curve analysis (DCA) by calculating the net benefit (NB) over a range of threshold probabilities.

As a sensitivity analysis, disease stage was examined *post hoc* to determine whether it contributed to predictive information beyond the pre-specified predictors. Model comparison was based on discrimination, information criteria, a likelihood ratio test for the added terms as a block, and the stability of the pre-specified predictor estimates.

### Establishment of clinical cutoff point

To facilitate clinical classification, a prevalence-based predicted probability threshold was selected using the observed event rate of active PTB in the study cohort. The corresponding linear predictor cutoff was calculated using the logit transformation of the selected probability threshold. Diagnostic performance, including sensitivity, specificity, positive predictive value (PPV), and negative predictive value (NPV), was evaluated across candidate probability thresholds, with the primary results reported at the prevalence-based threshold.

### Ethics approval and trial registration

This study was registered with the Thai Clinical Trials Registry (TCTR; ID: TCTR20260402004). Ethical approval was obtained from the Institutional Review Board of Buddhachinaraj Phitsanulok Hospital, Phitsanulok (IRB/HREC No. 002/2569), and the Human Research Ethics Committee of Naresuan University (Approval No. P3-0016/2569). The requirement for informed consent was waived due to the retrospective observational design. The study was conducted in accordance with the ethical standards of the responsible institutions and the Declaration of Helsinki.

## Results

Of 514 patients with HNC referred for radiotherapy, nine were excluded (six with prevalent active TB and three who withdrew before treatment initiation), leaving 505 eligible patients. Active PTB was diagnosed in 30 patients (5.94%), and 475 (94.06%) had no active PTB (Fig. 1).

**Figure 1:**
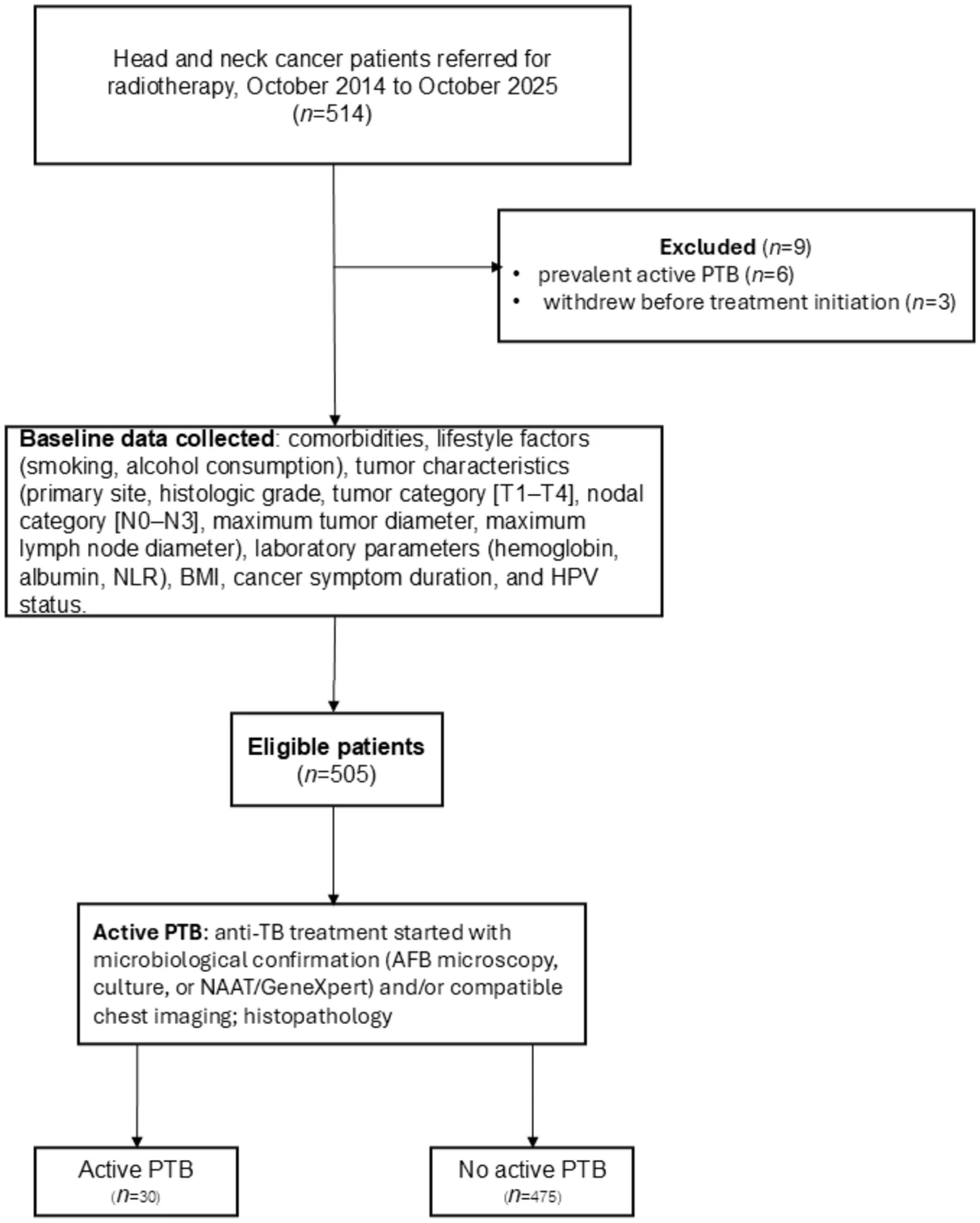
Study flow diagram of eligible patients with head and neck cancer.

### Baseline characteristics of patients with and without active PTB

The proportion of male patients did not differ significantly between the active PTB and no active PTB groups (30.0% vs. 44.6%; P = 0.131), nor did mean age (59.8 ± 11.4 vs. 57.3 ± 11.4 years; P = 0.234). Diabetes mellitus was significantly more common in patients with active PTB (16.7% vs. 6.3%; P = 0.048). Compared to the no active PTB group, patients with active PTB exhibited significantly lower BMI (17.3 ± 3.2 vs. 20.3 ± 3.8 kg/m^2^; P < 0.001), hemoglobin (9.9 ± 1.4 vs. 11.9 ± 2.0 g/dL; P < 0.001), and albumin (3.2 ± 0.5 vs. 3.8 ± 0.5 g/dL; P < 0.001) levels. The cancer symptom duration was significantly longer in the active PTB group (4.3 ± 2.4 vs. 2.3 ± 1.8 months; P < 0.001) (Table 1).

**Table 1:** Baseline Characteristics of Patients With and Without Active PTB

Baseline characteristics	Active PTB (n = 30), n (%)	No active PTB (n = 475), n (%)	P-value	AuROC
Sex, male, n (%)	9 (30.0)	212 (44.6)	0.131	0.573
Age (years), (mean ± SD)	59.8 ± 11.4	57.3 ± 11.4	0.234	0.544
Comorbidities				
Diabetes mellitus	5 (16.7)	30 (6.3)	0.048	0.551
Hypertension	4 (13.3)	78 (16.4)	0.802	0.515
Dyslipidemia	0 (0)	40 (8.4)	0.156	0.500
Smoking history	19 (63.3)	260 (54.7)	0.450	0.543
Alcohol consumption	19 (63.3)	250 (52.6)	0.266	0.553
Tumor site				
Larynx	8 (26.7)	63 (13.3)	0.082	0.643
Oropharynx	7 (23.3)	167 (35.2)		
Hypopharynx	10 (33.3)	98 (20.6)		
Oral cavity	3 (10.0)	83 (17.5)		
Nasopharynx	2 (6.7)	64 (13.5)		
Histologic grade				
Well differentiated	9 (30.0)	205 (43.2)	0.326	0.575
Moderately differentiated	16 (53.3)	192 (40.4)		
Poorly differentiated	5 (16.7)	78 (16.4)		
Tumor stage (T-stage)				
T1	0 (0)	38 (8.0)	0.165	0.580
T2	5 (16.7)	121 (25.5)		
T3	9 (30.0)	140 (29.5)		
T4	16 (53.3)	176 (37.1)		
Nodal stage (N-stage)				
N0	5 (16.7)	68 (14.3)	0.054	0.625
N1	3 (10.0)	121 (25.5)		
N2	15 (50.0)	239 (50.3)		
N3	7 (23.3)	47 (9.9)		
Hemoglobin (g/dL), mean ± SD	9.9 ± 1.4	11.9 ± 2.0	< 0.001	0.781
Albumin (g/dL), mean ± SD	3.2 ± 0.5	3.8 ± 0.5	< 0.001	0.812
HPV status (positive)	1/5 (20.0)	16/143 (11.2)	0.462	0.544
NLR, median (IQR)	3.9 (2.1, 7.0)	3.3 (2.1, 5.3)	0.338	0.552
Maximum tumor diameter (cm), mean ± SD	4.9 ± 1.7	4.6 ± 2.1	0.483	0.557
Maximum lymph node diameter (cm), Median (IQR)	2.0 (1.2, 5.0)	1.8 (1.0, 3.0)	0.169	0.563
Unresectable disease	28 (93.3)	419 (88.2)	0.560	0.520
BMI (kg/m^2^), (mean ± SD)	17.3 ± 3.2	20.3 ± 3.8	< 0.001	0.724
Cancer symptom duration (months), mean ± SD	4.3 ± 2.4	2.3 ± 1.8	< 0.001	0.793

Albumin was missing in 80 patients. HPV testing was performed mainly in patients with oropharyngeal tumors. Percentages are calculated among those with available HPV results (n = 148). AuROC: area under the receiver operating characteristic curve; BMI: body mass index; HPV: human papillomavirus; IQR: interquartile range; NLR: neutrophil-to-lymphocyte ratio; PTB: pulmonary tuberculosis; SD: standard deviation.

### Final model development: univariable and multivariable logistic regression analyses for active PTB

The four pre-specified predictors were entered simultaneously into the multivariable logistic regression model. The resulting conditional associations with active PTB were BMI (mOR: 0.86; 95% CI: 0.75–0.99), hemoglobin (mOR: 0.75; 95% CI: 0.58–0.95), albumin (mOR: 0.25; 95% CI: 0.12–0.53), and cancer symptom duration (mOR: 1.49; 95% CI: 1.25–1.76) (Table 2).

**Table 2:** Univariable and Multivariable Logistic Regression Analyses for Active PTB

Predictors	Univariable OR (95% CI)	P-value	Multivariable OR (95% CI)	P-value	AuROC
BMI (kg/m^2^)	0.76 (0.67, 0.87)	< 0.001	0.86 (0.75, 0.99)	0.047	0.882
Hemoglobin (g/dL)	0.60 (0.49, 0.74)	< 0.001	0.75 (0.58, 0.95)	0.021	
Albumin (g/dL)	0.14 (0.07, 0.29)	< 0.001	0.25 (0.12, 0.53)	< 0.001	
Cancer symptom duration (months)	1.41 (1.23, 1.63)	< 0.001	1.49 (1.25, 1.76)	< 0.001	

Multivariable odds ratios were estimated from a single model including body mass index, hemoglobin concentration, serum albumin concentration, and cancer symptom duration; each odds ratio is adjusted for the other three variables in the model. AuROC: area under the receiver operating characteristic curve; BMI: body mass index; CI: confidence interval; OR: odds ratio.

### Sensitivity analysis: contribution of nodal stage

Adding nodal stage to the four-predictor model produced only a marginal change in discrimination (AuROC 0.898 vs. 0.882) and did not improve model fit after accounting for the additional parameters (Akaike Information Criterion (AIC) 170.4 vs. 169.8; Bayesian Information Criterion (BIC) 204.2 vs. 190.9). Nodal stage was not statistically significant as a block (likelihood ratio test χ^2^(3) = 5.34, P = 0.149). The mORs of the four pre-specified predictors were essentially unchanged, with all shifts below 5%, and all four retained statistical significance. The four-predictor model was therefore retained.

### Apparent and internal validation performance of the prediction model

The final model demonstrated excellent discriminative ability, achieving an AuROC of 0.882 (95% CI: 0.814–0.950) (Fig. 2). The model exhibited excellent calibration, with an apparent calibration slope of 1.000 and a CITL of 0.000 (Fig. 3). Internal validation using bootstrap resampling indicated minimal optimism, C-statistic of 0.872 and shrinkage factor of 0.941 (Table 3).

**Figure 2:**
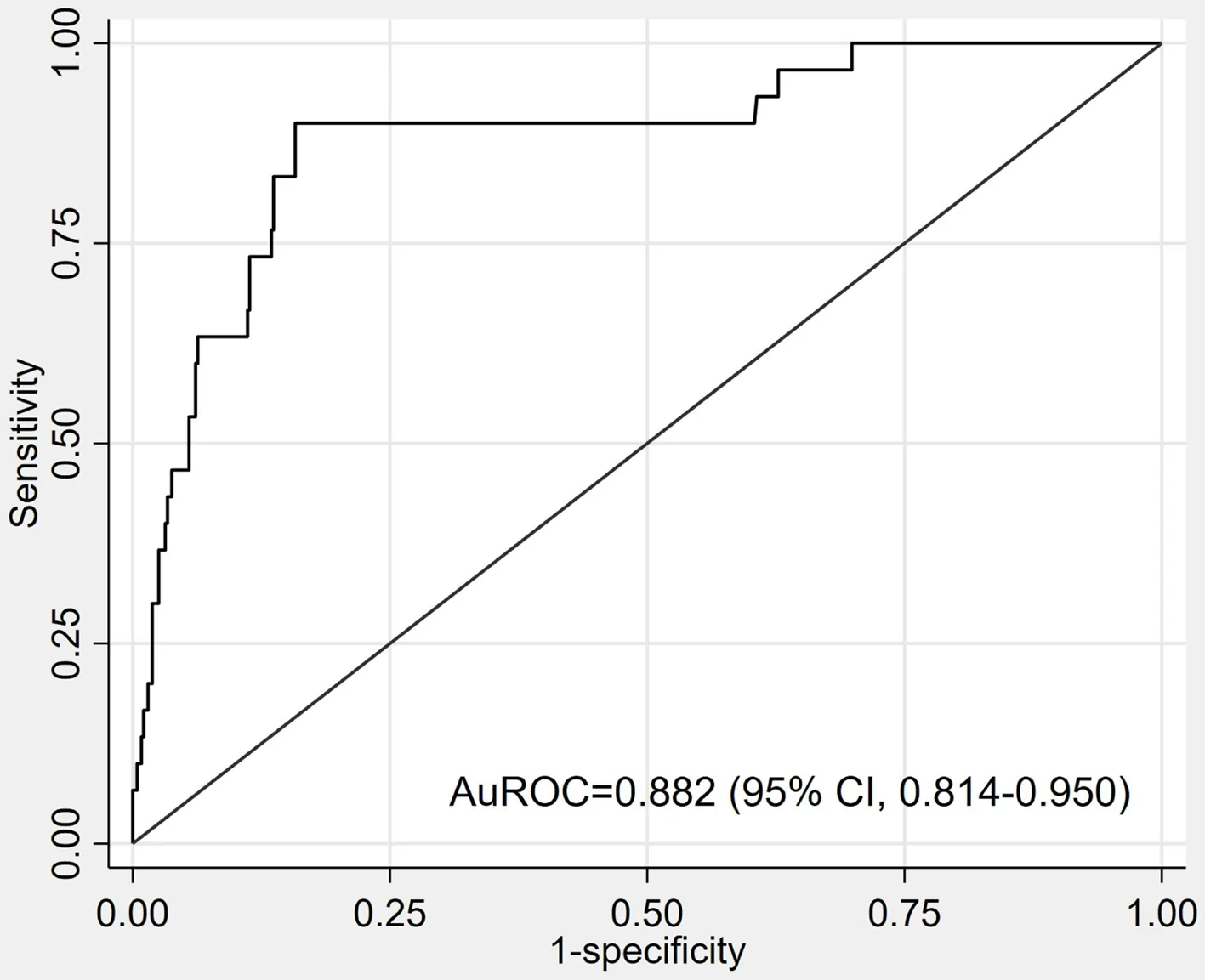
Receiver operating characteristic curve of the prediction model.

**Figure 3:**
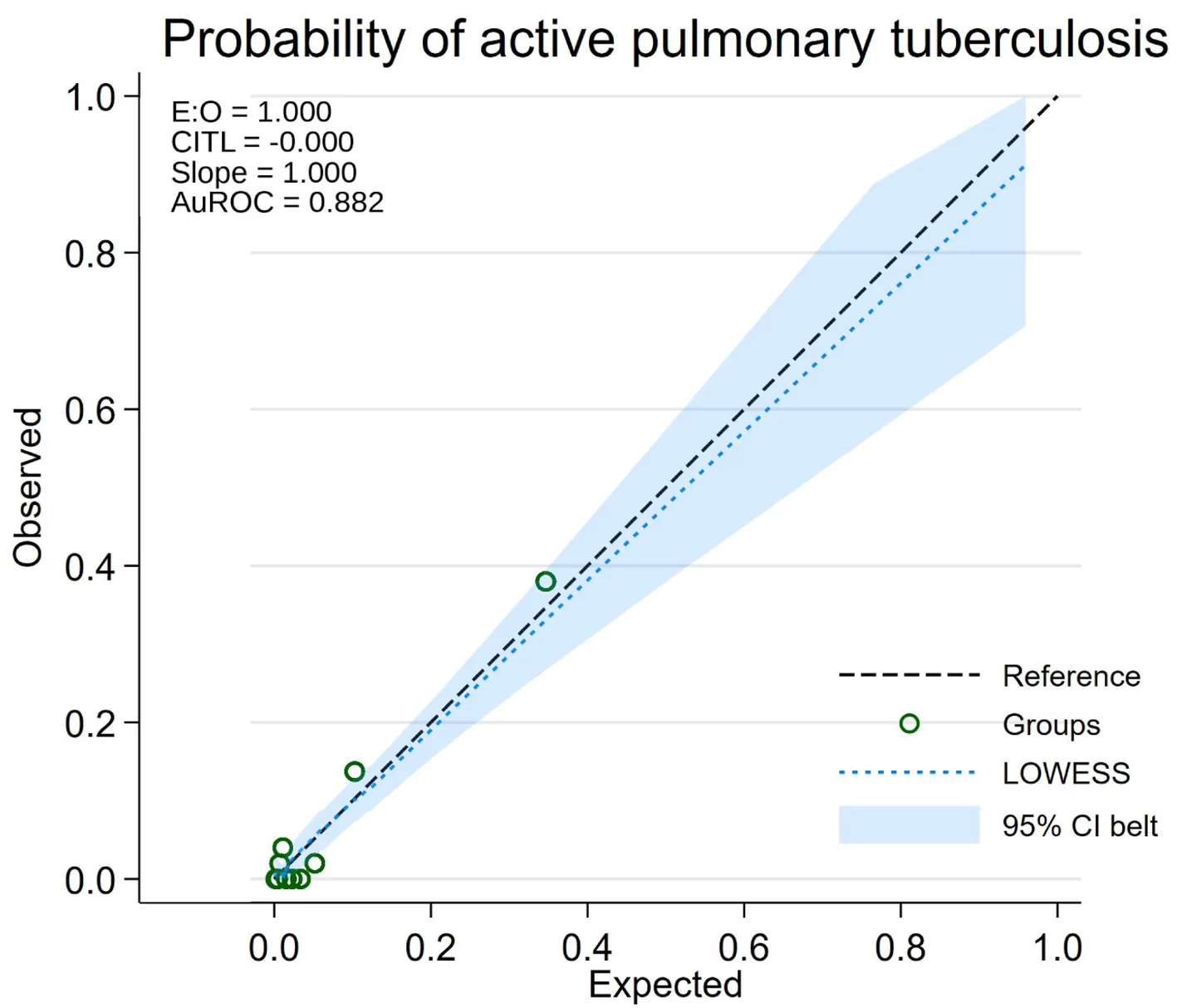
Calibration plot of the prediction model.

**Table 3:** Apparent and Internal Validation Performance of the Prediction Model

Parameters	Apparent performance (95% CI)	Bootstrap performance (95% CI)
C-statistic (AuROC)	0.882 (0.814, 0.950)	0.872 (0.805, 0.954)
E:O ratio	1.000	1.034 (0.712, 1.372)
CITL	0.000 (−0.421, 0.421)	−0.024 (−0.512, 0.536)
Calibration slope	1.000 (0.713, 1.287)	0.917 (0.668, 1.287)
Bootstrap shrinkage factor		0.941

AuROC: area under the receiver operating characteristic curve; CITL: calibration-in-the-large; E:O ratio: expected-to-observed outcomes ratio.

### Diagnostic performance of the prediction model at the prevalence-based threshold

Using a predicted probability threshold of 0.0594, the model achieved a sensitivity of 90.0% (95% CI: 73.5–97.9) and a specificity of 82.3% (95% CI: 78.6–85.6). At this cutoff, 27 of the 30 active PTB cases were correctly classified as high-risk. The model yielded three false negatives (0.6% of all patients) and 84 false positives (16.6% of all patients) (Table 4).

**Table 4:** Diagnostic Performance of the Prediction Model at the Prevalence-Based Threshold

Model probability of active PTB	Predicted Classification	Active PTB (n = 30), n (% of total)	No active PTB (n = 475), n (% of total)	Diagnostic metrics, % (95% CI)
≥ 5.94%	Positive	27 (5.3)	84 (16.6)	Sensitivity: 90.0% (73.5, 97.9) PPV: 24.3% (16.4, 33.7)
< 5.94%	Negative	3 (0.6)	391 (77.4)	Specificity: 82.3% (78.6, 85.6) NPV: 99.2% (97.8, 99.8)

CI: confidence interval; NPV: negative predictive value; PPV: positive predictive value; PTB: pulmonary tuberculosis.

DCA suggested the clinical utility of the model despite the low event rate, showing a higher NB than the treat-all and treat-none strategies across a range of clinically plausible threshold probabilities (Fig. 4). The observed prevalence of active PTB was 5.94% (30/505); therefore, the prevalence-based predicted probability threshold was set at 0.0594. This threshold corresponded to a linear predictor cutoff of −2.762. Accordingly, patients with a linear predictor ≥ −2.762, corresponding to a predicted probability ≥ 5.94%, were classified as high risk for active PTB (Fig. 5).

**Figure 4:**
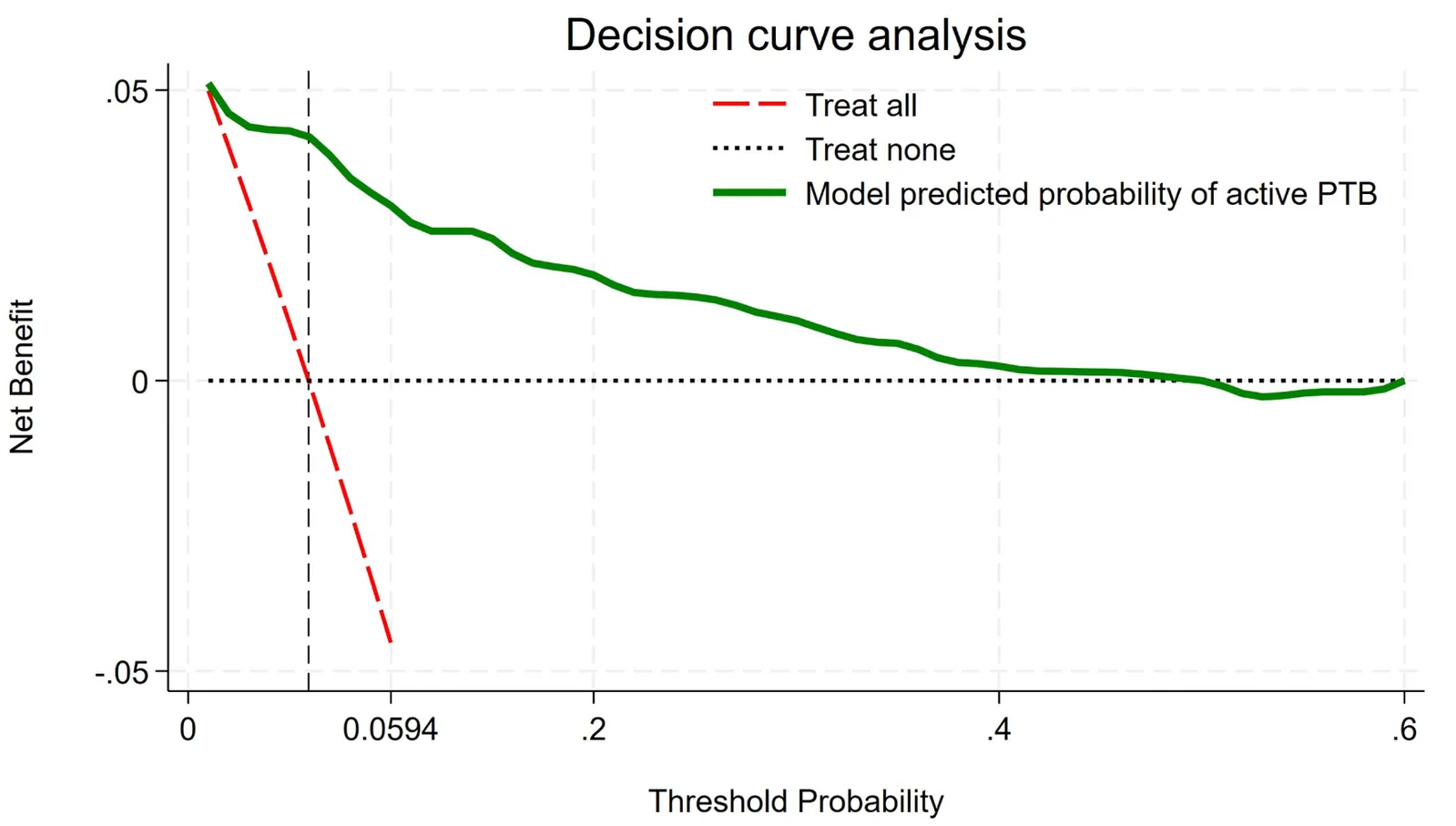
Decision curve analysis showing net benefit across threshold probabilities.

**Figure 5:**
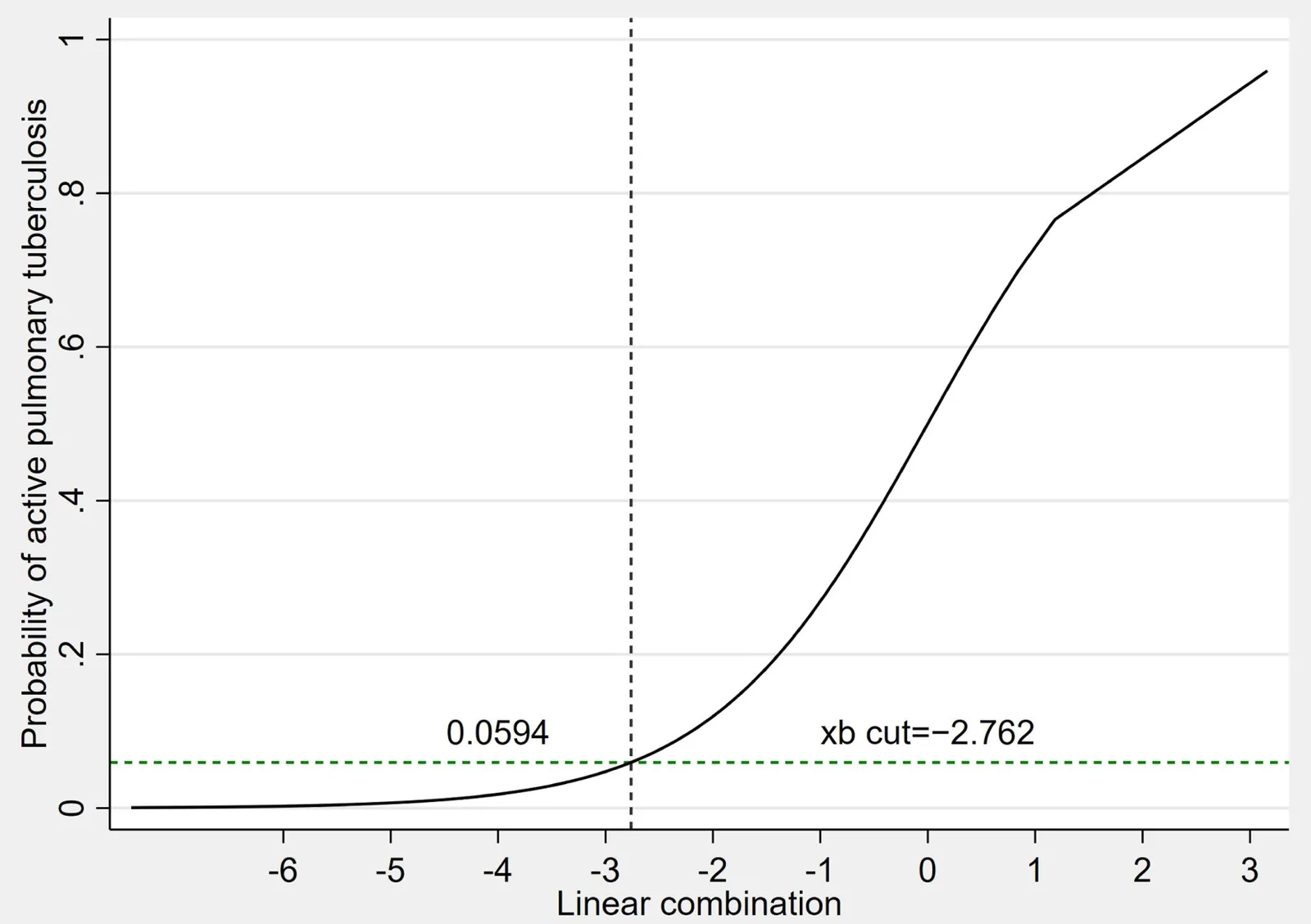
Relationship between the linear predictor and predicted probability of active PTB. PTB: pulmonary tuberculosis.

## Discussion

In this study, we report the prevalence of active PTB detected during pretreatment evaluation among patients with HNC undergoing radiotherapy. This may reflect the high proportion of locally advanced disease and prolonged cancer symptom duration in patients referred to radiotherapy. Our four-factor multivariable logistic regression model (BMI, hemoglobin, albumin, and cancer symptom duration) exhibited excellent discrimination and calibration. By applying a predicted probability cutoff based on the disease prevalence in the study cohort (5.94%), the sensitivity and specificity were 90.0% and 82.3%, respectively. This strategy may reduce unnecessary confirmatory active PTB workups while maintaining a low false-negative rate (0.6%). These results support a pragmatic triage tool that relies on routinely assessed clinical and laboratory variables to prioritize PTB screening before starting radiotherapy in resource-limited settings.

Biologically plausible features, such as low BMI, which was a strong predictor in our model, are known risk factors for active PTB and have been associated with an inverse association of the same. Large epidemiologic studies have shown a clear and log-linear association between BMI and the incidence of TB, especially within the range of 18.5–30 kg/m^2^; specifically, the risk of disease increases multiplicatively as BMI decreases [20]. This susceptibility is likely mediated, at least in part, by undernutrition-associated defects in host immune defenses, specifically the cell-mediated immunity needed to contain mycobacterial infection [20]. Clinically, these results underline the importance of heightened clinical suspicion and prompt PTB assessment during pretreatment staging in patients with HNC, as demonstrated in underweight or cachectic patients, particularly those exhibiting tumor-related dysphagia. Pretreatment albumin was a low-cost, readily available clinical surrogate in our model that reflected the baseline nutritional status and systemic inflammation. In other immunocompromised populations, the prognostic value of albumin for active PTB reactivation is well established; baseline hypoalbuminemia in patients starting antiretroviral therapy is a strong, independent predictor of incident PTB and overall mortality, adding further information to that provided by traditional immune markers such as CD4 cell counts [21]. Similarly, hypoalbuminemia in patients with HNC likely represents the combined impact of cancer-associated cachexia, chronic inflammation related to tumor burden, and poor oral intake. These processes cumulatively serve to drastically reduce physiological reserves and compromise cell-mediated immune responses, which are critical for containing mycobacterial infections. Anemia is associated with active PTB, supporting the inclusion of hemoglobin as a predictor in our model. A recently developed diagnostic risk prediction model [16] identified anemia as an independent predictor of active PTB, consistent with our findings. Mechanistically, anemia can be a symptom of chronic inflammation, micronutrient deficiencies, and impaired immune function, pathways that are significantly exaggerated in patients with HNC. This increased susceptibility can be attributed to tumor-related systemic inflammation, malnutrition, and cancer-associated cachexia, which drive the progression from latent to active PTB.

The risk of TB was most substantial within the first year following a cancer diagnosis and was higher among patients receiving chemotherapy or radiotherapy, suggesting a time-dependent susceptibility related to treatment-induced immunosuppression [7]. These findings are biologically plausible, as oncologic treatments can disrupt mucosal and structural barriers and cause profound immunosuppression, thereby facilitating the progression from latent TB infection to active disease. These findings highlight the importance of early PTB assessment during pretreatment staging, before the initiation of immunosuppressive oncologic treatment.

Another study developed a nomogram based on clinical history and symptoms, specifically underlying disease, TB contact history, maximum temperature, and weight loss. This model demonstrated strong discrimination in both the derivation (AuROC 0.810) and validation (AuROC 0.864) cohorts [19]. Although the model was designed to support empirical diagnosis when microbiological confirmation is unavailable or delayed, it specifically focuses on differentiating PTB from community-acquired pneumonia (CAP) in children. While the target population differed from that in our study, certain predictors, such as weight loss, are conceptually similar to BMI used in our model, as both serve as indicators of nutritional status. A primary-care PTB prediction model demonstrated acceptable discrimination (AuROC 0.82) and calibration (slope 0.98; intercept 0.001), making it suitable as a screening and triage tool to reduce missed cases and prioritize limited confirmatory testing, such as GeneXpert [16]. Because the PTB prevalence in that study was higher (17%) and the objective was population-level screening in resource-limited settings, model performance and optimal thresholds may differ when applied to lower-prevalence populations or cohorts with different symptom profiles. Nevertheless, its overall discrimination and calibration were comparable to those in our study.

Biomarker-based machine learning (ML) approaches for the diagnosis of TB perform substantially differently than other algorithms. Well-performing models (for example, probabilistic neural networks) resulted in very high accuracy (sensitivity 96.1%, specificity 89.9%, AuROC 0.94), but decision tree models had an advantage in sensitivity (95.2%) at the expense of low specificity (58.7%) [22]. These differences are objective in their clinical utility: high-sensitivity models are appropriate for triage (rule-out), whereas high-specificity approaches are necessary to reduce false positives prior to confirmatory testing. However, the clinical use of biomarker-based ML is often constrained by costs, infrastructure requirements, and a lack of standardization. In contrast, our model is based on routinely available clinical and laboratory data. It demonstrated good discrimination and calibration, offering a practical, low-cost alternative that may reduce the proportion of patients requiring confirmatory active PTB testing.

Patients with solid tumors are often immunosuppressed; however, they may have a lower cumulative lifetime risk of TB reactivation than other high-risk populations. Consequently, clinical guidelines remain controversial; while some infectious disease societies support latent TB infection (LTBI) screening and preventive therapy for patients receiving systemic cancer treatment, comprehensive oncology guidelines often lack specific consensus [3]. A major contributor to this conflict is the limited evidence, particularly regarding LTBI screening outcomes and prophylaxis in specific malignancies, such as HNC. Therefore, defining the incidence and timing of active PTB is necessary to prevent the disease burden, identify high-risk subgroups, and inform targeted screening strategies.

### Limitations

This study has several limitations. First, the inclusion period spanned 11 years (October 2014 to October 2025), during which TB diagnostic practices and clinical care evolved and which also encompassed the COVID-19 pandemic, potentially affecting TB services and cancer referral patterns. Temporal effects were not formally assessed because the limited number of active PTB events precluded reliable period-specific model fitting or adequately powered predictor-by-period interaction analyses. The temporal stability and transportability of the model should therefore be evaluated in temporally distinct and more contemporary cohorts.

Second, disease stage was not included among the pre-specified predictors. In a *post hoc* sensitivity analysis, adding nodal stage did not provide sufficient incremental predictive value to justify the additional model complexity in this cohort. Whether T or N stage provides additional predictive information beyond the four pre-specified predictors should be evaluated in larger cohorts with a greater number of active PTB events.

Third, comorbidities were ascertained from pretreatment medication records rather than standardized diagnostic criteria, and smoking and alcohol exposure were recorded as binary ever-versus-never variables without quantitative information on intensity or duration. Although these variables were not included in the final prediction model, the limited granularity of these data prevented a more detailed assessment of their potential predictive contribution.

Finally, the model was derived from a single tertiary radiotherapy center in a TB-endemic setting, was based on only 30 active PTB events, and has undergone internal validation only. External validation in independent populations, particularly in settings with different TB prevalence, patient case mix, and diagnostic capacity, is required before the model can be recommended for routine clinical use.

### Conclusion

We developed a four-variable clinical prediction model utilizing routinely available pretreatment parameters—BMI, hemoglobin, albumin, and cancer symptom duration—to accurately estimate the probability of active PTB in patients with HNC. This model performed well in terms of discrimination and calibration, representing an inexpensive risk assessment tool that can be implemented to support clinical decision-making in resource-limited and TB-endemic settings worldwide. This underscores the urgent need to maintain a high index of clinical suspicion and standardized risk assessments that would facilitate timely TB evaluation by oncology teams.

## Data Availability

The datasets generated and analyzed during this study are not publicly available due to patient privacy restrictions but can be obtained from the corresponding author upon reasonable request.

## Abbreviations

AFB: acid-fast bacilli
AuROC: area under the receiver operating characteristic curve
BMI: body mass index
CAP: community-acquired pneumonia
CI: confidence interval
CITL: calibration-in-the-large
CT: computed tomography
DCA: decision curve analysis
E:O ratio: expected-to-observed ratio
HIV: human immunodeficiency virus
HNC: head and neck cancer
HPV: human papillomavirus
IQR: interquartile range
IRB: Institutional Review Board
LTBI: latent tuberculosis infection
MDT: multidisciplinary team
ML: machine learning
mOR: multivariable odds ratio
NAAT: nucleic acid amplification testing
NLR: neutrophil-to-lymphocyte ratio
NPV: negative predictive value
OR: odds ratio
PPV: positive predictive value
PTB: pulmonary tuberculosis
SD: standard deviation
TB: tuberculosis
TCTR: Thai Clinical Trials Registry
